# Placebo Responses Among Men With Erectile Dysfunction Enrolled in Phosphodiesterase 5 Inhibitor Trials: A Systematic Review and Meta-analysis

**DOI:** 10.1001/jamanetworkopen.2020.1423

**Published:** 2020-03-20

**Authors:** Alexander Stridh, Moa Pontén, Stefan Arver, Irving Kirsch, Christoph Abé, Karin B. Jensen

**Affiliations:** 1 Department of Clinical Neuroscience, Karolinska Institute, Solna, Sweden; 2 Department of Medicine, Karolinska Institute, Solna, Sweden; 3 Program in Placebo Studies, Beth Israel Deaconess Medical Center, Harvard Medical School, Boston, Massachusetts

## Abstract

**Importance:**

Placebo responses in the treatment of erectile dysfunction (ED) are poorly described in the literature to date.

**Objective:**

To quantify the association of placebo with ED outcomes among men enrolled in placebo-controlled, phosphodiesterase 5 inhibitor (PDE5I) trials.

**Data Sources:**

For this systematic review and meta-analysis, a database search was conducted to identify double-blind, placebo-controlled studies using PDE5Is for the treatment of ED published from January 1, 1998, to December 31, 2018, within MEDLINE, Embase, Cochrane Library, and Web of Science. Only articles published in the English language were included.

**Study Selection:**

Double-blind, placebo-controlled randomized clinical trials of PDE5Is for ED were included. Studies were excluded if they did not provide distribution measures for statistical analysis. Study selection review assessments were conducted by 2 independent investigators. A total of 2215 studies were identified from the database search, and after review, 63 studies that included 12 564 men were analyzed.

**Data Extraction and Synthesis:**

Preferred Reporting Items for Systematic Reviews and Meta-Analyses (PRISMA) guidelines were followed in abstracting data and assessing validity. Data were extracted from published reports by 2 independent reviewers. Quality assessment was performed using the Jadad scale. Data were pooled using a random-effects model.

**Main Outcomes and Measures:**

The main outcome was improvement in the erectile function domain of the International Index of Erectile Function questionnaire in the placebo arm of the included studies. Effect size was reported as bias-corrected standardized mean difference (Hedges *g*). The hypothesis was formulated before data extraction.

**Results:**

A total of 63 studies that included 12 564 men (mean [SD] age, 55 [7] years; age range, 36-68 years) were included. Erectile function was significantly improved among participants in the placebo arm, with a small to moderate effect size (Hedges *g* [SE], 0.35 [0.03]; *P* < .001). Placebo effect size was larger among participants with ED associated with posttraumatic stress disorder (Hedges *g* [SE], 0.78 [0.32]; *P* = .02) compared with the overall analysis. No significant difference was found between placebo and PDE5Is for ED after prostate surgery or radiotherapy (Hedges *g* [SE], 0.30 [0.17]; *P* = .08).

**Conclusions and Relevance:**

In this study, placebo was associated with improvement of ED, especially among men with ED-related posttraumatic stress disorder. No difference was found between placebo and PDE5I among men treated for ED after prostate surgery.

## Key Points

**Question:**

Was there an association between placebo and improved erectile function among men enrolled in studies of phosphodiesterase 5 inhibitors?

**Findings:**

This systematic review and meta-analysis of 12 564 men found a significant association between placebo and improved erectile function, with the effect size being larger among men with posttraumatic stress disorder. There was no difference between response to placebo and phosphodiesterase 5 inhibitors in men with erectile dysfunction after prostate surgery.

**Meaning:**

The findings suggest that contextual factors are important in the delivery of care to patients with sexual dysfunction, and the lack of difference in response between placebo and phosphodiesterase 5 inhibitors in certain patient subgroups suggests that clinical practice should change.

## Teaser

This systematic review and meta-analysis assesses whether there is an association between placebo and erectile dysfunction outcomes among men enrolled in placebo-controlled, phosphodiesterase 5 inhibitor trials.

## Introduction

In the past few decades, few drugs have achieved the same mythologic status as sildenafil. Approved in 1998 for treatment of erectile dysfunction (ED), it was soon accompanied by tadalafil and vardenafil, and more recently a few additional drugs within the class of phosphodiesterase 5 inhibitors (PDE5Is) entered the market. Sildenafil was originally developed to relieve symptoms of angina pectoris. What was first considered an adverse effect turned out to be beneficial for increasing blood flow in other areas as well.^1^ Anecdotes from early clinical trials describe study participants being unwilling to return unused pills because of the positive erectile effects of the study drug. Despite its specific effect on blood flow through relaxing the penile cavernosal smooth muscle cells, the reputation of the drug has become synonymous with potency, increased virility, and improved sexual performance in general. A previous study^2^ reported that sildenafil and other equivalent drugs have been used recreationally by men (both adults and adolescents) without ED. Given the reputation of this class of drugs, it seems possible that some of the effects of the drugs may be related to the power of belief.

Erectile function can be divided into a central component that influences the sympathetic outflow from the thoracolumbar region of the spinal cord and a peripheral reflexogenic erectile function mediated by nitrergic nerves that project from the sacral region of the spinal cord.^3^ Erectile dysfunction can be caused by many adversities, such as cardiovascular disease, diabetes, smoking, hypogonadism, iatrogenesis due to pelvic surgery, and adverse effects of medication, but it can also be of psychogenic origin. It has been estimated that approximately 80% of ED is peripheral in origin, although psychogenic factors is likely associated with ED in these cases too. The severity of ED is commonly diagnosed using the International Index of Erectile Function questionnaire (IIEF).^4,5^ A previous study^6^ performed in the United States estimated the prevalence of ED to be 44% to 70% in men 60 years and older, with increased prevalence with advancing age. A European study^7^ has estimated that ED prevalence ranges from 6% to 64% beginning at 40 years of age and becoming more prevalent in the upper range of the age span.

In any clinical trial, improvement in the placebo arm is common. Placebo effects have been demonstrated in many conditions, such as Parkinson disease, pain disorders, anxiety disorders, depression, asthma, and irritable bowel syndrome.^8^ Several neurobiological mechanisms have been proposed to underlie placebo effects, including involvement of endogenous opioids,^9,10^ endogenous cannabinoids,^11^ and activation of dopaminergic neurons.^12,13^ The placebo effect has also been demonstrated in neuroimaging studies,^14,15^ which have found increased neural activations in brain structures involved in reduction of, for example, pain or motor symptoms.

We conducted a systematic review and meta-analysis on the use of PDE5Is for ED. The primary goal was to quantify the change in erectile function among patients in the placebo arm of randomized clinical trials as measured by the erectile function domain of the IIEF questionnaire. To our knowledge, this is the first comprehensive meta-analysis that quantifies the association of placebo with ED outcomes in randomized clinical trials of PDE5I.

## Methods

### Data Sources and Searches

The review protocol was preregistered in the PROSPERO database for meta-analyses (CRD42018109553), including a full account of searches, inclusion and exclusion criteria, main outcomes, and an analysis plan. This study followed the Preferred Reporting Items for Systematic Reviews and Meta-analyses (PRISMA) reporting guideline. Data were obtained in collaboration with the Karolinska Institute Library. Searches in MEDLINE, Embase, Cochrane Library, and Web of Science Core Collection were performed for all randomized clinical trials published between January 1, 1998, and December 31, 2018, that focused on PDE5Is for ED treatment.

The most common measure to evaluate erectile function is the erectile function domain of the IIEF (IIEF-EF). Along with the IIEF-EF, the other domains of the IIEF are orgasmic function (IIEF-OF), intercourse satisfaction (IIEF-IS), sexual desire (IIEF-SD), and overall satisfaction (IIEF-OS). The IIEF-EF domain has a top score of 30, with a score below 14 indicating clinically impaired ED function and recommending use of a PDE5I.^4,5^ A change of 4 points or more is considered clinically meaningful. The IIEF-EF domain was chosen as the primary outcome of this meta-analysis. Some studies used the abridged 5-question version of the IIEF, Sexual Health Inventory for Men.

### Study Selection

**Figure 1.**
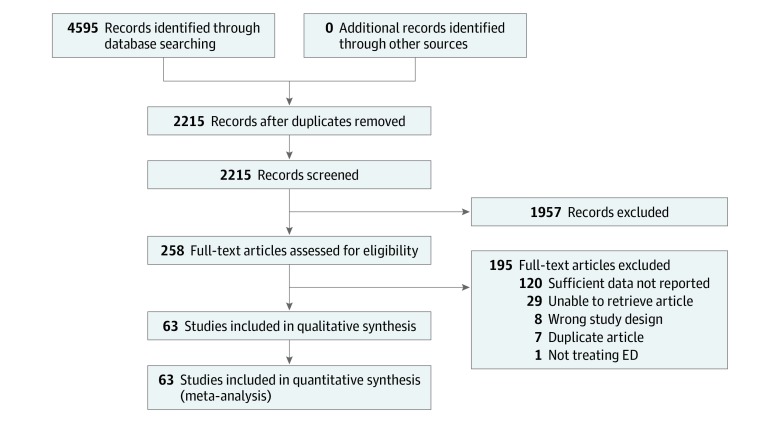
Flowchart for the Trials Included in the Meta-analysis ED indicates erectile dysfunction.

The selection of studies to be included in our analysis was independently conducted by 2 of us (A.S. and C.A.) in a blinded fashion using the Rayyan software for meta-analyses (Qatar Computing Research Institute). The inclusion criteria were double-blind, placebo-controlled randomized clinical trials that used PDE5I for ED treatment or had ED as a comorbidity for another condition and that were reported in English. After unblinding with the Rayyan software, any discrepancies between the 2 reviewers were reconciled in a consensus meeting. The study selection procedure adhered to the Consolidated Standards of Reporting Trials (CONSORT) reporting guideline to ensure adequate quality of included studies. A flowchart of study selection is given in Figure 1.

### Statistical Analysis

Data extraction was independently conducted by 2 of us (A.S. and M.P.). Studies were excluded from the meta-analysis if they did not contain any IIEF questionnaire data, did not report any separate data for drug and placebo arms, provided incomplete data, did not allow the calculation of an effect size, or used mixed treatments with proposed effects for ED in the treatment regimen (eg, testosterone). The quality of included studies was graded with Jadad scores^16^ and was included in the risk of bias assessments at the study and outcome levels.

The Comprehensive Meta-Analysis software, version 3.0, was used for data management and statistical calculations of bias-corrected standardized mean differences (Hedges *g*). Effect size is commonly interpreted as small (Hedges *g*, 0.2), moderate (Hedges *g*, 0.5), or large (Hedges *g*, 0.8). Treatment response data from various domains of the IIEF questionnaire were analyzed separately per treatment arm as measured from before to after treatment, as well as the difference in outcomes between patients receiving active treatment and those receiving placebo. All analyses were performed with a random-effects approach using a 2-tailed α = . 05.

Studies that contained arms with different doses of active drug treatment were averaged to a mean treatment response. In line with a prespecified protocol, subanalyses were performed to explain effect size heterogeneity. Moderators included mean age of participants, study duration, evidence of financial interest, study drug, Jadad scale (range of 0-5, with higher numbers indicating higher quality), and comorbidities. A random-effect metaregression analysis was conducted on mean age of study participants, study drug, study duration, and influence of financial interest by study sponsor or investigators.

## Results

**Figure 2.**
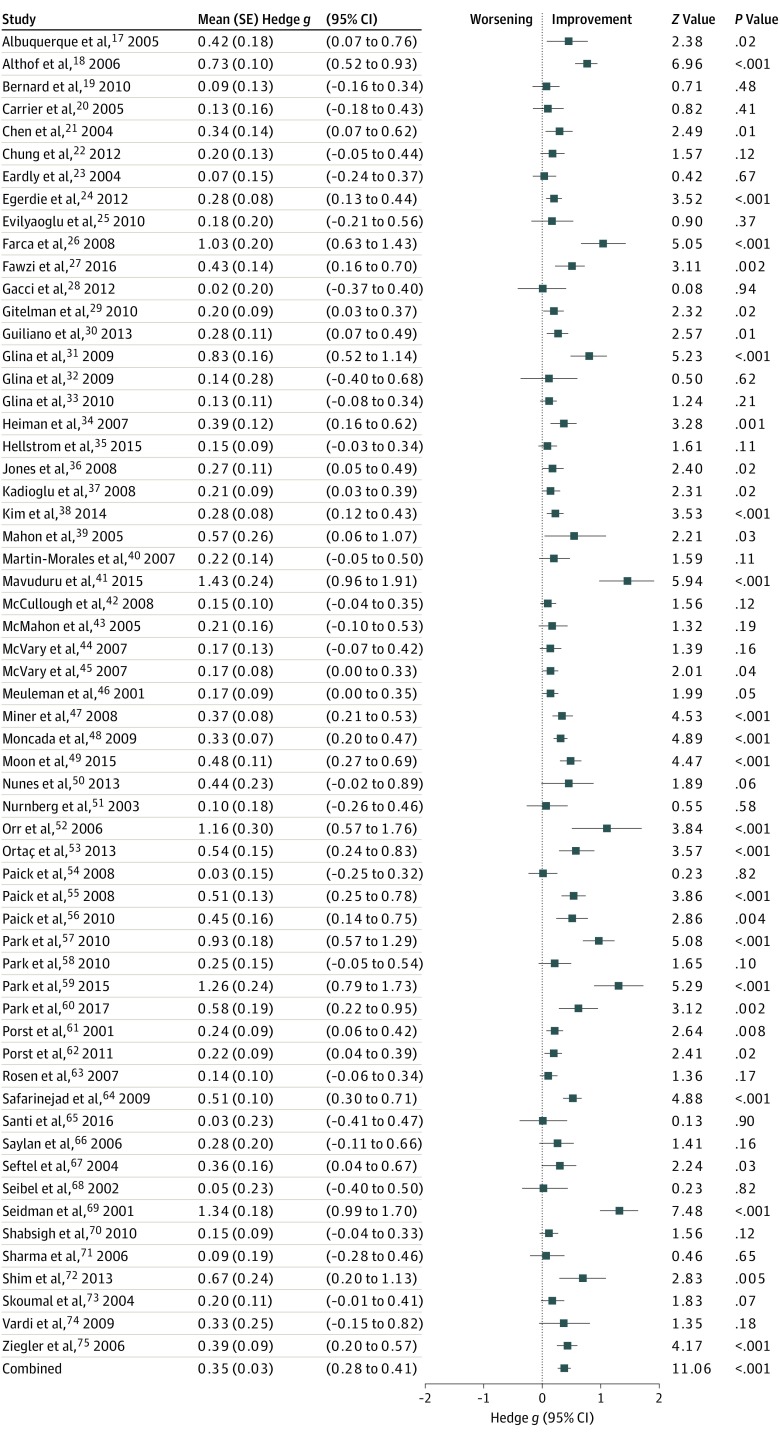
Forest Plot of the Association Between Placebo and Erectile Disfunction Outcomes Erectile dysfunction outcomes were measured using the Erectile Function Domain of the International Index of Erectile Function. A low to moderate improvement was seen in the placebo arm, as indicated by the bias-corrected standardized mean difference (Hedges *g* [SE], 0.35 [0.03]; *P* < .001).

A total of 63 studies fulfilled the inclusion criteria; 59 studies were included in the main analysis that assessed the effect size before and after treatment for the various domains of IIEF. Four studies were analyzed separately because PDE5Is were used for treatment of ED after prostate cancer treatment and the design and patient groups differed considerably from the other studies (end of treatment scores indicate worsened erectile function compared with baseline because of surgery or radiotherapy). The treatment effect size in this subset of studies was calculated in a traditional drug vs placebo comparison. The methodologic quality of studies had a mean (SD) of 3.6 (0.88) points on the Jadad scale.^16^ A funnel plot was created in the Comprehensive Meta-Analysis software and revealed no risks of publication bias. The 63 included trials (Table 1) included a total of 12 564 men with ED (mean [SD] age, 55 [7] years; age range, 36-68 years). Some studies reported the cause of ED, but in general, studies represented a mix of peripheral and centrally mediated causes of ED, which was commonly referred to in the literature as organic and psychogenic. The mean duration of a treatment trial was 14 weeks (range, 4-104 weeks). A calculation on the combined effect size within the drug and placebo arms was conducted for all included studies in the main analysis (*k* = 59) excluding the 4 studies in patients with prostate surgery. The effect size in the placebo arm showed a small to moderate improvement of erectile function (Hedges *g* [SE], 0.35 [0.03]; *I*^2^ = 70.48; *P* < .001). The overall effect size in the drug arm showed a large response (Hedges *g* [SE], 1.25 [0.07]; *I*^2^ = 93.34; *P* < .001). A comparison between responses in the drug and placebo arms revealed a large difference in favor of active drug (Hedges *g* [SE], 1.04 [0.08]; *I*^2^ = 92.38; *P* < .001) (Figure 2).

**Table 1.** Studies Included in the Meta-analysis

Source	Study drug	No. of patients	Jadad score	Financial interest	Mean age, y	Study duration, wk
Albuquerque et al,^17^ 2005	Sildenafil	87	2	Yes	60.0	8
Althof et al,^18^ 2006	Sildenafil	282	3	Yes	55.0	12
Bernard et al,^19^ 2010	Sildenafil	162	3	Yes	50.0	8
Carrier et al,^20^ 2005	Tadalafil	239	4	Yes	59.0	12
Chen et al,^21^ 2004	Tadalafil	194	4	Yes	59.5	12
Chung et al,^22^ 2012^a^	Mirodenafil	134	3	Yes	55.5	12
Eardly et al,^23^ 2004	Tadalafil	215	3	Yes	53.5	12
Egerdie et al,^24^ 2012	Tadalafil	583	4	Yes	62.5	12
Evilyaoglu et al,^25^ 2010	Tadalafil	50	3	No	42.5	12
Farca et al,^26^ 2008	Sildenafil	89	3	Yes	56.0	12
Fawzi et al,^27^ 2016^a^	Sildenafil	131	5	No	66.0	24
Gacci et al,^28^ 2012^a^	Vardenafil	59	5	No	67.0	12
Gitelman et al,^29^ 2010	Vardenafil	327	3	Yes	62.0	12
Guiliano et al,^30^ 2013	Tadalafil	211	3	Yes	63.0	12
Glina et al,^31^ 2009	Sildenafil	129	3	Yes	53.5	12
Glina et al,^32^ 2009	Lodenafil	60	4	Yes	54.5	4
Glina et al,^33^ 2010	Lodenafil	319	4	Yes	55.5	4
Heiman et al,^34^ 2007	Sildenafil	176	5	Yes	58.0	12
Hellstrom et al,^35^ 2015	Avanafil	414	3	Yes	58.0	8
Jones et al,^36^ 2008	Sildenafil	202	3	Yes	52.0	10
Kadioglu et al,^37^ 2008	Sildenafil	294	2	Yes	45.0	6
Kim et al,^38^ 2014	Tadalafil	592	4	Yes	58.0	12
Mahon et al,^39^ 2005	Sildenafil	16	3	Yes	53.0	12
Martin-Morales et al,^40^ 2007	Vardenafil	121	3	Yes	53.0	12
Mavuduru et al,^41^ 2015^a^	Tadalafil	82	4	No	NA	4
McCullough et al,^42^ 2008	Sildenafil	260	3	Yes	52.5	8
McMahon et al,^43^ 2005	Tadalafil	133	5	Yes	59.5	26
McVary et al,^44^ 2007	Tadalafil	156	3	Yes	61.5	12
McVary et al,^45^ 2007	Sildenafil	351	4	Yes	60.0	12
Meuleman et al,^46^ 2001	Sildenafil	315	3	Yes	54.5	26
Miner et al,^47^ 2008	Vardenafil	386	3	No	54.5	12
Moncada et al,^48^ 2009	Sildenafil	817	3	Yes	56.0	12
Moon et al,^49^ 2015	Udenafil	346	5	Yes	58.5	24
Nunes et al,^50^ 2013	Lodenafil	48	5	No	37.5	8
Nurnberg et al,^51^ 2003	Sildenafil	77	5	Yes	45.0	6
Orr et al,^52^ 2006	Sildenafil	42	4	Yes	45.0	4
Ortaç et al,^53^ 2013	Udenafil	118	5	Yes	43.5	8
Paick et al,^54^ 2008	Udenafil	164	3	Yes	55.0	12
Paick et al,^55^ 2008	Mirodenafil	222	3	Yes	53.5	12
Paick et al,^56^ 2010	Mirodenafil	107	3	Yes	57.5	12
Park et al,^57^ 2010	Udenafil	103	3	Yes	53.0	4
Park et al,^58^ 2010	Mirodenafil	108	2	Yes	56.5	12
Park et al,^59^ 2015^a^	Udenafil	73	5	No	55.0	12
Park et al,^60^ 2017	Avanafil	158	4	Yes	56.5	8
Porst et al,^61^ 2001	Vardenafil	580	4	Yes	52.0	12
Porst et al,^62^ 2011	Tadalafil	300	3	Yes	65.0	12
Rosen et al,^63^ 2007	Vardenafil	216	4	Yes	58.0	8
Safarinejad et al,^64^ 2009	Sildenafil	242	4	No	48.0	4
Santi et al,^65^ 2016	Vardenafil	42	5	No	55.5	24
Saylan et al,^66^ 2006	Tadalafil	132	3	Yes	50.5	12
Seftel et al,^67^ 2004	Tadalafil	205	4	Yes	59.0	12
Seibel et al,^68^ 2002	Sildenafil	41	5	No	47.5	4
Seidman et al,^69^ 2001	Sildenafil	136	3	Yes	56.0	12
Shabsigh et al,^70^ 2010	Sildenafil	266	3	Yes	40.0	8
Sharma et al,^71^ 2006	Sildenafil	32	4	No	40.0	8
Shim et al,^72^ 2013^a^	Udenafil	49	3	No	60.0	8
Skoumal et al,^73^ 2004	Tadalafil	403	3	Yes	52.0	12
Vardi et al,^74^ 2009	Sildenafil	53	2	Yes	55.0	4
Ziegler et al,^75^ 2006	Vardenafil	302	4	Yes	50.3	12
Prostate cancer trials						
Zelefsky et al,^76^ 2014^a^	Sildenafil	181	2	Yes	NA	104
Incrocci et al,^77^ 2001	Sildenafil	60	3	Yes	68.0	12
Padma-Nathan et al,^78^ 2008	Sildenafil	76	5	Yes	56.0	48
Pisansky et al,^79^ 2014	Tadalafil	96	4	Yes	63.0	52

Abbreviation: NA, not available.

^a^ Studies that used the abridged 5-question version of the International Index of Erectile Function (Sexual Health Inventory for Men).

An analysis was performed on the other domains of the IIEF (ie, the IIEF-OF, IIEF-IS, IIEF-SD, and IIEF-OS). Not all included studies reported data on all domains; therefore, the number of studies per domain in this follow-up analysis was reduced. All results of IIEF subdomains are given in Table 2. For the effect size on orgasmic function (IIEF-OF; n = 31), the drug arm showed a high response, whereas the placebo arm showed a lower response, and between-group analysis showed a moderate response in favor of the drug arm. For intercourse satisfaction (IIEF-IS; n = 40), the drug arm showed a large response, the placebo arm showed a moderate response, and the between-group analysis showed a large effect size that favored the study drug. For sexual desire (IIEF-SD; n = 30), the drug arm showed a moderate response, and the placebo arm showed a low to moderate response. The between-group analysis was in favor of the study drug, with a moderate effect size. For overall satisfaction (IIEF-OS; n = 39), the drug arm showed a large response, whereas the placebo arm showed a low to moderate response. The between-group analysis showed a large effect size that favored the drug arm.

**Table 2.** Results From All IIEF Survey Domains Other Than Erectile Function^a^

Domain	Studies, No.	Hedges *g,* (SE)	*P* value
Intercourse satisfaction (IIEF-IS)			
Drug arm	40	1.27 (0.09)	<.001
Placebo arm	40	0.52 (0.05)	<.001
Between	40	0.88 (0.08)	<.001
Overall satisfaction (IIEF-OS)			
Drug arm	39	1.17 (0.08)	<.001
Placebo arm	39	0.35 (0.04)	<.001
Between	39	0.89 (0.09)	<.001
Sexual desire (IIEF-SD)			
Drug arm	30	0.60 (0.06)	<.001
Placebo arm	30	0.25 (0.25)	<.001
Between	30	0.40 (0.04)	<.001
Orgasmic function (IIEF-OF)			
Drug arm	31	0.75 (0.05)	<.001
Placebo arm	31	0.21 (0.03)	<.001
Between	31	0.57 (0.05)	<.001

Abbreviations: IIEF, International Index of Erectile Function; IS, intercourse satisfaction; OF, orgasmic function; OS, overall satisfaction; SD, sexual desire.

^a^ Data are given in descending order of effect size in the placebo arm. Not all studies used all subscales, which accounts for the different number of studies. Effect sizes are reported as bias-corrected standardized mean difference (Hedges *g*).

The effect size for studies in which ED was associated with posttraumatic stress disorder (PTSD; n = 2) indicated a large response for the drug arm (Hedges *g* [SE], 1.12 [0.39]; *I*^2^ = 77.79; *P* = .004) and a large response in the placebo arm (Hedges *g* [SE], 0.77 [0.32]; *I*^2^ = 76.15; *P* = .02). The between-group analysis yielded a moderate effect size in favor of the study drug (Hedges *g* [SE], 0.40 [0.17]; *I*^2^ = 27.64; *P* = .02).

Using a regression model, we assessed the association of moderators with ED treatment responses. In an overall analysis including data from the drug and placebo arms combined, no significant association of PDE5I drug type with treatment responses was found (*q* = 10.02; *df* = 6; *I*^2^ = 94.44; *P* = .12). The association was significant when the placebo arm was analyzed separately (*q* = 15.96; *df* = 6; *I*^2^ = 68.63; *P* = .01), but no significant association was seen in the drug arm (*q* = 11.14; *df* = 6; *I*^2^ = 92.83; *P* = .08) or in the between-group comparisons (*q* = 11.79; *df* = 6; *I*^2^ = 92.71; *P* = .07). There was a high correlation between treatment responses in the drug and placebo arm (*r* = 0.67), and without avanafil, a drug assessed in only 2 of the 63 studies, the correlation was higher (*r* = 0.94). There was no significant association of any of the other moderators with ED treatment responses (ie, financial interest, study duration, mean study participant age, or Jadad score). For these moderators, there were no significant associations for drug and placebo combined, for drug and placebo arms separately, or in a traditional drug vs placebo comparison.

An explorative analysis was conducted on the studies using PDE5Is as aid in recovery of erectile function after prostate surgery or radiotherapy (n = 4). This between-group analysis of the effect size did not show a significant response in favor of drug vs placebo (Hedges *g* [SE], 0.29 [0.17]; *I*^2^ = 59.35; *P* = .08).

## Discussion

This study found a significant association between placebo treatment and IIEF-EF scores in patients with ED, but the potential mechanisms are still unexplored. One possibility is that the association between placebo effect and erectile function are mediated by an increased nervous tone in the thoracolumbar tract because this nerve tract is mainly influenced by arousal mechanisms.^3^ Several neurobiological mechanisms have been proposed to underlie placebo effects. Endogenous opioids and cannabinoids have been proposed to mediate the placebo effect in various conditions.^9,10,11^ Given that these 2 substrates (opioid and cannabinoid) are mainly involved in a negative association with sexual arousal, it seems unlikely that they are associated with the placebo effect in treatment for ED. Another commonly proposed neural substrate for the placebo effect, the dopaminergic system,^13^ would be more likely to be involved in the association between placebo effect and erectile function because dopamine has a positive association with sexual arousal.^80^ The dopaminergic hypothesis is supported by dopamine agonists having been used in the treatment of ED.^81,82,83^

There was a significant association of the active drug with erectile function scores in patients with ED. Given that the site of action of PDE5Is is the smooth muscle cells that influence the blood flow necessary to achieve erection, it seems plausible that the effect of the active drug would vary depending on the cause of the ED. If the problem were mainly vascular or endocrinologic, such as in atherosclerosis, diabetes, or hypogonadism, it seems plausible that the PDE5Is would have a strong effect because they address the underlying pathophysiological cause. If the nerves to the penis have been severed or severely damaged (as in the 4 prostate cancer trials in this analysis), the PDE5Is cannot amplify the nervous signal to the smooth muscle cells; thus, PDE5Is would have no specific effect.

Other domains of the IIEF questionnaire showed variations in effect sizes, in which responses in the drug arm were lower for orgasmic function (IIEF-OF) and sexual desire (IIEF-SD) compared with erectile function (IIEF-EF). This finding was expected because orgasmic function and especially sexual desire are associated with numerous factors other than the ability to achieve erection. There were also variations in effect size in the placebo arm, in which the response was lower for IIEF-OF and IIEF-SD compared with IIEF-EF. This finding was most likely attributable to the same factors as the differences among domains in the drug arm. Of interest, the response for intercourse satisfaction (IIEF-IS) was higher compared with the IIEF-EF response in the placebo arm. This result was not concordant with the result in the drug arm, and because the degree of the association with intercourse satisfaction may be more affected by psychological factors, this result supports separate mechanisms for placebo and drug improvements in patients with ED.

The 2 studies^52,64^ on treatment for PTSD-associated ED had a lower effect size for the drug response compared with results from the main analysis. The effect size of the placebo response in PTSD studies was markedly higher compared with the main analysis. Assuming that ED in this patient group was associated with psychological stress caused by traumatic experiences, the large improvement in the placebo arm could indicate that psychological factors might be associated with ED symptom improvements. This finding is supported by clinical studies^84,85^ that found comparable ED improvements for psychological interventions and PDE5Is, or increased improvements when combined (compared with PDE5Is alone).^86^ However, neither the drug nor placebo arm in the 2 studies^52,64^ on ED associated with PTSD reached a median IIEF-EF score, indicating normal erectile function, although clinically significant improvements were observed.

A metaregression analysis revealed a significant association between PDE5I drug type and treatment responses in the placebo arm and high correlation between the treatment response in the drug and placebo arms. The association of PDE5I drug type with erectile function in individuals given placebo suggests that differences in subjective perception of the different drugs exist because they differ in brand names, marketing, and visual appearance. Previous research shows that placebo effects are associated with labels^87^ and marketing,^88^ and it is possible that such differences contributed to the results in the present study.

No significant association of study duration, participant age, Jadad score, or financial interest with treatment effect size was found in the drug or placebo arm. Study duration is difficult to compare with previous studies of placebo longevity because PDE5Is are taken when needed, and the effects may be different from placebo responses in long-term use of, for example, antidepressants.^89^ Furthermore, there was little variation in participant age among studies, and new studies are needed to determine whether placebo responses in ED treatment may differ depending on participant age. The lack of an association between documented financial interests and treatment responses suggests that the trials included in this meta-analysis were not biased by the potential influence of a study sponsor.

The Jadad score reflects the quality of the clinical trials included in the meta-analysis. The lack of an association between Jadad scores and treatment outcomes indicates that the results in this meta-analysis are not biased by study quality.

Usually, PDE5Is are only taken before sexual intercourse. It has been theorized that daily long-term treatment with PDE5Is after prostate surgery and radiotherapy can aid the healing of damaged nerves through an amplification of the nervous input to the smooth muscles of the cavernous body.^90^ Previous research^4^ regarding the usefulness of this practice has been inconclusive. The results from the present meta-analysis showed no statistically significant differences between the response in the drug and placebo arms among patients who underwent treatment for prostate cancer. Individual studies^91,92^ that are not part of this meta-analysis have found stronger associations of erectile function and active drug compared with placebo. However, differences in the degree of nerve damage in the pelvic area may represent a confounding factor by which patients with less severe nerve damage after surgery or radiotherapy might receive a short-term benefit from PDE5Is (unrelated to the proposed effects of long-term treatment). The results in this meta-analysis suggest that PDE5Is have no significant association with the recovery of erectile function after prostate surgery or radiotherapy. Until robust differences between drug and placebo have been demonstrated, the practice of prescribing daily intake of PDE5Is after prostate cancer treatment may not be considered evidence, questioning the long-term use of PDE5Is for these patients.

### Limitations

This study has limitations. This meta-analysis is limited by the inability to compare improvements in the placebo arm with no-treatment data from the included study populations. In any placebo-controlled drug trial, the inclusion of a no-treatment group will help understanding of how much of the treatment response is attributable to the drug itself, how much is attributable to the placebo effect, and how much of the treatment response is attributable to factors such as spontaneous remission and regression to the mean. We cannot exclude that the observed placebo response was partly associated with spontaneous ED improvements. The inclusion of a no-treatment control group in randomized clinical trials of ED is warranted because it would potentially lead to a better understanding of placebo effects in PDE5I treatment trials.

## Conclusions

This systematic review and meta-analysis found a significant association of placebo and ED outcomes, with larger effect sizes among men with PTSD-associated ED. No difference in erectile function was found between those who received placebo vs PDE5I for ED after prostate surgery.
